# Interaction between *Akkermansia muciniphila* and Diet Is Associated with Proinflammatory Index in School-Aged Children

**DOI:** 10.3390/children10111799

**Published:** 2023-11-10

**Authors:** Juan Carlos Ayala-García, Alba Mariel García-Vera, Alfredo Lagunas-Martínez, Yaneth Citlalli Orbe-Orihuela, Ana Cristina Castañeda-Márquez, Cinthya Estefhany Díaz-Benítez, Víctor Hugo Bermúdez-Morales, Miguel Cruz, Margarita Bahena-Román, Ana Isabel Burguete-García

**Affiliations:** 1 Centro de Investigación Sobre Enfermedades Infecciosas, Instituto Nacional de Salud Pública, Cuernavaca 62100, Mexico; carlos.ayala@insp.edu.mx (J.C.A.-G.); alagunas@insp.mx (A.L.-M.); yaneth.orbe@insp.mx (Y.C.O.-O.); cediaz@insp.mx (C.E.D.-B.); vbermudez@insp.mx (V.H.B.-M.); mbahena@insp.mx (M.B.-R.); 2 Escuela de Salud Pública de México, Instituto Nacional de Salud Pública, Cuernavaca 62100, Mexico; mariel.garcia@insp.edu.mx; 3 Instituto de Investigación Científica, Universidad Juárez del Estado de Durango, Durango 34100, Mexico; cristy_acm@hotmail.com; 4 Unidad de Investigación Médica en Bioquímica, Centro Médico Nacional Siglo XXI, Ciudad de México 06720, Mexico; miguel.cruzlo@imss.gob.mx

**Keywords:** gut microbiota, dietary patterns, cytokines, inflammation

## Abstract

Background: Imbalance in the intestinal microbiota can lead to chronic low-grade inflammation. Diet may influence this association. In this study, we aimed to evaluate the interaction between *Akkermansia muciniphila* (*A. muciniphila*) and dietary patterns using a proinflammatory index. Methods: We conducted a cross-sectional study with school-aged children. We quantified the relative abundance (RA) of *A. muciniphila* in feces using a polymerase chain reaction. We collected dietary information through employing a food frequency questionnaire and generated dietary patterns using principal component analysis. We generated a proinflammatory index from serum levels of interleukin-6, interleukin-10, tumor necrosis factor alpha, and adiponectin validated by receptor operating characteristic curves. We evaluated the association between *A. muciniphila* and the proinflammatory index using logistic regression, including an interaction term with dietary patterns. Results: We found that children with a low RA of *A. muciniphila* and a high intake of simple carbohydrates and saturated fats had increased odds of being high on the proinflammatory index. However, when the consumption of this dietary pattern is low, children with a low RA of *A. muciniphila* had decreased odds of being high on the proinflammatory index. Conclusions: Our results suggest that the simultaneous presence of *A. muciniphila* and diet have a more significant impact on the presence of being high on the proinflammatory index compared to both factors separately.

## 1. Introduction

From birth, the human intestine is colonized by millions of viruses, protozoa, fungi, and mainly bacteria. This balanced ecosystem is called the intestinal microbiota and plays a fundamental role in the maintenance of human health [1]. One bacterium, *Akkermansia muciniphila* (*A. muciniphila*), has gained prominence for its health-promoting properties. *A. muciniphila* is a mucin-degrading bacterium of the phylum *Verrucomicrobia* that resides in the mucous layer of the intestinal lining [2,3]. This bacterium feeds on mucin, a glycoprotein secreted by the intestinal lining to protect and lubricate the mucosal surface, thus contributing to the maintenance of a healthy mucosal layer that supports the integrity of the intestinal barrier [4]. Several studies have described the association between the abundance of *A. muciniphila* with improved metabolic health, reduced body weight, increased insulin sensitivity, and reduced risk of obesity, as well as its anti-inflammatory properties, relevant for the treatment of chronic inflammatory diseases [5,6,7,8].

The proportion, richness, and diversity of the intestinal microbiota depend mainly on the diet and the availability of substrates for microbial fermentation. Different dietary patterns can lead to the predominance of specific bacterial species, affecting the usual composition of the microbiota (intestinal dysbiosis) [9]. It has been established in the literature that *A. muciniphila* predominates in an environment rich in mucin-derived carbohydrates and fibers, such as those found in plant-based diets. The fermentation of these fibers results in the production of short-chain fatty acids (SCFAs), such as acetate, propionate, and butyrate [10]. These SCFAs are a vital energy source for the intestinal epithelium, particularly butyrate, which exerts an anti-inflammatory effect by reducing the expression of proinflammatory genes [11].

Inflammation is an essential physiological response for host defense and tissue repair. However, when inflammation becomes chronic, it can contribute to the development of numerous diseases, such as metabolic syndrome, inflammatory bowel, and cardiovascular diseases [12,13]. *A. muciniphila* has been noted for its potential to reduce chronic inflammation through multiple mechanisms. First, it helps to reinforce the mucosal layer in which it resides by feeding on mucin. This helps to regulate the thickness and stability of the mucosal barrier, decreasing the risk of intestinal permeability [14]. Secondly, the extracellular vesicles of this microorganism regulate tight junction proteins that strengthen the barrier function in the intestinal lining, which also helps to reduce intestinal permeability [15]. Finally, *A. muciniphila* can influence the response of the immune system by increasing the production of regulatory T cells and decreasing the production of proinflammatory cytokines, such as tumor necrosis factor alpha (TNF-α) and interleukin-6 (IL-6) [16,17]. Therefore, in this study, we aimed to evaluate the interaction between intestinal *A. muciniphila* and dietary patterns using a proinflammatory index in Mexican school-aged children.

## 2. Materials and Methods

### 2.1. Design and Study Population

A cross-sectional design was conducted with data from a previous investigation [18]. We included unrelated children living in four areas of Mexico City (Cuauhtémoc, Morelos, Independencia, and Nezahualcóyotl). Those diagnosed with infectious diseases, gastrointestinal disorders, and under antibiotic treatment two months before the beginning of the study were excluded. Data from 1006 children aged 6 to 12 years were analyzed, with complete information on the relative abundance (RA) of *A. muciniphila*, frequency of food intake, serum cytokines, and other covariates of interest.

### 2.2. Relative Abundance of Akkermansia muciniphila

We obtained 200 mg of stool samples to extract bacterial DNA using the QIAmp^®^ Fast DNA Stool mini kit (Qiagen, Hilden, Germany). We determined DNA concentration and purity (260/280 nm) using the Thermo Scientific™ Lite NanoDrop™ spectrophotometer (Madison, WI, USA). We used the quantitative polymerase chain reaction (q-PCR) technique to amplify a variable region of the 16S ribosomal RNA gene of *A. muciniphila* on the StepOnePlus™ Real-Time PCR System (Woodlands, Singapore).

Each amplification was performed in duplicate to reduce the margin of error in amplification efficiency, and a negative control was placed on each plate. The q-PCR was performed under the following conditions: an initial denaturation step of 10 min at 95 °C, denaturation for 15 s at 95 °C, alignment at 58 °C (Universal) and 63 °C (*A. muciniphila*) for 15 s, and extension at 95 °C for 1 min.

Finally, for the dissociation curve, the conditions were 95 °C for 15 s, 60 °C for 1 min, and a gradual temperature increase of 0.1/0.2 °C until reaching 95 °C. The specific amplification of the gene of interest was confirmed by dissociation curves, verifying that the fluorescence over time (dF/dT) showed a single peak.

To carry out the reaction, we used 5 µL of 2× Maxima SYBR Green/ROX qPCR Master Mix (2×) Thermo Scientific™ (Carlsbad, CA, USA), 1 µL of forward oligonucleotide (2×) Thermo Scientific™, 1 µL of forward oligonucleotide (CAGCACGTGAAGGTGGGGAC), 1 µL of reverse oligonucleotide (CCTTGCGGTTGGCTTCAGA) [19], 2 μL of Fermentas^®^ nucleic acid-free water (Carlsbad, CA, USA), and 1 μL of the DNA sample (10 ng/μL of *A. muciniphila*).

To normalize relative expression units, we used the universal forward oligonucleotide (AAACTCAAAAAKGAATTGACGG) and the universal reverse oligonucleotide (CTCACRRCACGAGCTCTGAC) [20]. The RA units were obtained using the comparative 2^−ΔCt^ method (*A. muciniphila* Ct—Universal Ct) [21].

### 2.3. Dietary Patterns

Based on Willet’s methodology, the information to identify dietary patterns was obtained through an adapted food frequency questionnaire (FFQ) for the study population [22]. This questionnaire is divided into ten sections (dairy products; fruits; vegetables; legumes; egg, meats, and deli; typical dishes; cereals; drinks, snacks, and desserts; fats; and supplements). It evaluates the frequency of intake of 111 foods with 10 response options ranging from “never” to “6 or more times a day” during the year before the application. According to the reported intake data, the average grams of food and total energy intake for each child in the study were calculated.

All the foods and drinks in this questionnaire were grouped into 33 food groups according to the similarity in their nutritional content. Total energy intake was examined to identify dietary patterns, removing results above the standard deviation. The average daily food intake was calculated according to the reported intake data. The mean obtained was multiplied by the grams contained in each portion of food and the total energy intake of each child. The foods were classified into groups according to the similarity of the nutrient content between them.

From the reported frequency, the percentage of the consumption of each food group contributing to the total daily diet was obtained. The z-scores were calculated for each food group and, subsequently, a principal component analysis with orthogonal rotation was performed. Patterns with an eigenvalue > 1.5 and factor loadings > 0.35 were retained. The result was the construction of the following two food patterns: Pattern 1, simple carbohydrates and saturated fats, and Pattern 2, complex carbohydrates and proteins (Table S1).

### 2.4. Proinflammatory Index

We collected blood samples via venipuncture after 12 h of fasting, and then the samples were centrifuged to obtain serum. The serum concentrations of TNF-α, IL-6, interleukin-10 (IL-10), and adiponectin were measured using enzyme-linked immunoadsorption assay kits according to the manufacturer’s instructions PeproTech^®^ (Rocky Hill, CT, USA). The absorbance readings were determined on a Labsystems Multiskan MS^®^ (Vantaa, Finland).

To generate the proinflammatory index, we used standardized serum concentrations of cytokines. First, we multiplied the standardized IL-10 and adiponectin values by (−1). Next, we added the standardized IL-6 and TNF-α values to the transformed standardized IL-10 and adiponectin values to create a proinflammatory index.

To evaluate the criterion validity of this index, we generated receiver operating characteristic curves to evaluate the new index created from the sum of cytokine z-scores with the gold standard (the concentrations of each of the cytokines Il-6, IL-10, TNF-α, and adiponectin). We confirmed that all the data obtained showed statistical significance (with a null value of 0.5), thus demonstrating the ability of this instrument to adequately identify children with inflammation (Table S2). Subsequently, we categorized this index according to our population median into high (≥to the median) and low (<to the median).

### 2.5. Data Collection

Through self-reporting, we collected information on personal pathological and non-pathological antecedents and a family history of type 2 diabetes (T2D), systemic arterial hypertension, and overweight–obesity. The questionnaire consisted of 18 questions applied by trained personnel to the parents or guardians of the children.

### 2.6. Anthropometry

Anthropometric measurements were collected using calibrated instruments by specialized personnel trained in the pediatric population. These measurements included weight, height, and body mass index (BMI).

### 2.7. Physical Activity

We obtained the metabolic equivalents (METs) per hour/week by applying the physical activity and inactivity questionnaire for schoolchildren in Mexico City (CAINM) [23]. The questionnaire collected information about activity and leisure activity during the last month. 

### 2.8. Statistical Analysis

We stratified our population according to their proinflammatory index level. We evaluated the normality of the continuous variables using the Shapiro–Wilk test. None of the continuous variables followed a normal distribution, so differences between groups were assessed using the Mann–Whitney U test. We used the chi-square test to explore differences between the groups for the categorical variables.

We generated tertiles of the RA of *A. muciniphila* and intake for each dietary pattern for the inferential analysis. We performed logistic regression models to evaluate the association between the RA of *A. muciniphila* and the tertiles of the intake of both dietary patterns with the proinflammatory index. In addition, to perform the interaction analysis, we employed logistic regression models, introducing an interaction term between the tertiles of the RA of *A. muciniphila* and the tertiles of the intake of each of the different daily patterns.

All models were adjusted for potential confounders identified in our directed acyclic graph. The minimum adjustment set included age, sex, physical activity, BMI, family history of overweight–obesity, and dietary patterns. We set statistical significance at *p* < 0.05 for the exploratory analysis and models without interaction. For models with interaction, statistical significance was established with a *p*-value < 0.10. All analyses were performed using Stata^®^ version 15 software.

## 3. Results

Table 1 presents the general characteristics of the population according to their proinflammatory index levels. Our population comprises 503 children who were high on the proinflammatory index and 503 children who were low on the proinflammatory index. Children who were high on the proinflammatory index had a higher median age, a higher prevalence of family history of overweight–obesity, higher BMI, and higher levels of IL-6 and TNF-α compared to those who were low on the proinflammatory index. In addition, we observed that the children who were low on the proinflammatory index presented higher concentrations of IL-10 and adiponectin compared to those who were high on the proinflammatory index. We found no statistically significant differences in sex distribution, family history of T2D, total energy intake, physical activity, and the RA of *A. muciniphila* between the two groups.

When evaluating the association between the RA of *A. muciniphila* and the proinflammatory index, we observed that children with a low and medium RA of *A. muciniphila* had decreased odds of being high on the proinflammatory index compared to children with a high RA of *A. muciniphila* [OR 0.86 (95% CI: 0.62, 1.18) and OR 0.84 (95% CI: 0.61, 1.16)]. However, this association was not statistically significant (Table 2).

Table 3 shows the association between dietary patterns and the proinflammatory index. We found that children with a medium intake of complex carbohydrates and protein had decreased odds of being high on the proinflammatory index [OR 0.92 (95% CI: 0.66, 1.27)]. On the other hand, children with a high intake of this same dietary pattern had increased odds of being high on the proinflammatory index than children with a low intake of complex carbohydrates and protein [OR 1.03 (95% CI: 0.75, 1.43)]. Also, we observed a reduction in the odds of being high on the proinflammatory index in children with medium and high intakes of simple carbohydrates and saturated fats compared with children with a low intake of this same dietary pattern [OR 0.85 (95% CI: 0.61, 1.18) and OR 0.83 (95% CI: 0.58, 1.15)]. No association was statistically significant.

Because we found no association between the RA of *A. muciniphila* and dietary patterns with the proinflammatory index, we considered exploring this interaction to evaluate the simultaneous effect of *A. muciniphila* and diet. We found no statistically significant association between the RA of *A. muciniphila* and complex carbohydrates and protein intake on the proinflammatory index. However, we did observe a reduction in the odds of presenting as high on the proinflammatory index among all levels of the RA of *A. muciniphila* with all levels of intake of this dietary pattern (Table 4).

Table 5 shows that children with a low RA of *A. muciniphila* and a low intake of simple carbohydrates and saturated fats had a 49% reduction in the odds of being high on the proinflammatory index compared to children with a high RA of *A. muciniphila* [OR 0.51 (95% CI: 0.29, 0.89)]. Also, we observed a 63% increase in the odds of being high on the proinflammatory index in children with a low RA of *A. muciniphila* and a high intake of simple carbohydrates and saturated fats compared to children with a high RA of *A. muciniphila* [OR 1.63 (95% CI: 0.90, 2.94)].

## 4. Discussion

Our main finding indicates that a low RA of *A. muciniphila* and a high intake of simple carbohydrates and saturated fats increase the odds of being high on the proinflammatory index [OR 1.63 (95% CI: 0.90, 2.94)]. These results are consistent with those obtained in a previous study of obese mice where oligofructose was administered as a prebiotic. These researchers reported increased *A. muciniphila* abundance and decreased obesity-related disorders induced by a high-fat diet [24]. These disorders are related to the presence of chronic low-grade inflammation.

Additionally, it has been observed that in mice with fatty liver disease, resulting from a high-fat diet, supplementation with inulin had the ability to reverse the accumulation of fat in the liver. After the administration of inulin to this group of mice, an increase from 10% to 47% in the presence of *A. muciniphila* was recorded [25]. This is because diets that are rich in inulin, a carbohydrate present in plants, and polysaccharides, promote the growth of *A. muciniphila* [26].

On the other hand, the reduction in proinflammatory cytokines may be related to decreased serum lipopolysaccharide (LPS) levels. This is supported by a study in overweight and obese individuals where the effects of supplementation with pasteurized *A. muciniphila* were investigated. The said study reported a significant reduction in plasma LPS levels (*p* = 0.019) [27]. The decrease in LPS in the blood prevents its binding to Toll-like receptor 4 (TLR-4), which, in turn, reduces the presence of cytokines in the blood [28]. The transition of LPS into the bloodstream occurs through the intestinal barrier, which relies on junctions that allow for the selective passage of molecules. Among the proteins responsible for these junctions are zonula occludens (ZO-1, 2, 3) and occludins. Any alteration in the function or stability of these tight junctions can compromise the integrity of the intestinal barrier, consequently increasing permeability [29].

*A. muciniphila* possesses a mechanism that contributes to improving the function of the intestinal barrier. This occurs through the production of SCFAs, which bind to FFAR3-FFAR2 receptors (also known as GPR43-GPR41). This interaction stimulates the secretion of glucagon-like peptides, such as GLP1 and GLP2. These peptides play a crucial role in regulating glucose metabolism and improving the function of the intestinal barrier [30].

In a state of optimal health, colonic epithelial cells utilize butyrate as an energy source through a beta-oxidation process in the mitochondria. This process involves oxygen consumption and contributes directly to maintaining anaerobic conditions in the intestinal lumen. In addition, butyrate binds to peroxisome proliferator-activated receptor gamma (PPARγ), which, in turn, suppresses inducible nitric oxide synthase (iNOS) activity. As a result, the production of nitric oxide (NO) and, ultimately, nitrate generation is reduced [28].

The ability of *A. muciniphila* to break down mucin and use it as its main source of carbon and nitrogen is of great importance. This process provides energy to both colonic epithelial cells and other coexisting microorganisms, such as *Anaerostipes caccae*, *Eubacterium hallii*, and *Faecalibacterium prausnitzii*, which employ the sugars generated from mucin degradation [26]. Mucin metabolism has the potential to stimulate the production of more mucin, increasing its thickness and strengthening the epithelial barrier in the intestine [31]. Mucins are large, highly glycosylated proteins that play a key role in protecting the interior of the intestine by reducing the potential translocation of proinflammatory lipopolysaccharides [24].

In this study, we observed a beneficial association between the relative abundance of *A. muciniphila* and a diet enriched in complex carbohydrates and protein. Although we did not reach a level of statistical significance, these results align with the findings of previous research in which mice were fed grape polyphenols, a dietary component characterized by a high complex carbohydrate and protein content. In that study, an increase in the abundance of *A. muciniphila* was recorded, which, in turn, contributed to strengthening the integrity of the intestinal lining [32].

One of the mechanisms that could explain this phenomenon is that *A. muciniphila* utilizes carbohydrates from mucin, composed of fucose, galactose, N-acetylgalactosamine (GalNAc), and N-acetylglucosamine (GlcNAc), and provides these substrates to other coexisting microorganisms [33]. Although it has not yet been determined whether *A. muciniphila* can break down natural polysaccharides, a positive relationship has been observed between the presence of *A. muciniphila* and the intake of dietary fiber oligosaccharides as well as the polysaccharides present in marine algae, such as *Enteromorpha clathrata*. Dietary components such as capsaicin, puerarin derived from *Pueraria lobate* root, mucosa-influencing proanthocyanidins from wild blueberry, apple flavonoids, hops, polyphenols from green tea, and soybean oil are some of the foods that have been associated with an increased presence of *A. muciniphila* [29].

One relevant finding in particular is related to the production of SCFAs, which is directly influenced by the type of fiber consumed. For example, it has been identified that inulin promotes propionate production, whereas resistant starches favor butyrate generation [34]. This observation could be linked to the consumption patterns of simple carbohydrates and saturated fats, as many individuals consume foods such as sweet bread, cookies, buns, and white bread, which all contain resistant starches.

This study presents various notable strengths. The first is that our evaluation of *A. muciniphila* was performed using qPCR following the international standards. Secondly, our proinflammatory index was constructed from serum levels of cytokines, which were measured using standardized laboratory procedures, greatly decreasing the possibility of measurement errors.

However, when interpreting the results, it is important to keep in mind some limitations of our study. The questionnaire used to collect information on diet in the year prior to the interview could have led to errors in the measurement due to the possible lack of precision in recalling the foods consumed by the children in our study. However, it is relevant to highlight that the questionnaire was uniformly applied to all participants by trained personnel, and information was collected in a consensual manner between caregivers and children, which guarantees the veracity of the data. Therefore, any errors that may have occurred are considered non-differential and are unlikely to affect the validity of the final results.

Regarding reverse causality, we consider that there is a low probability that this influences our results. The international literature shows that diet has the ability to modify the composition of the gut microbiota and subsequently affect the inflammatory response. Since our interaction analysis allows us to evaluate the joint effect of *A. muciniphila* and dietary patterns on the proinflammatory index, the causal pathways proposed in prospective studies are not compromised. In addition, we have evaluated a proinflammatory profile based on cytokine levels in children without metabolic, infectious, or inflammatory diseases, ensuring that the outcome does not precede exposure.

Finally, another limitation is that we do not have information on the use of probiotics. We know that the use of probiotics, as well as the amount and type, could influence the composition of the gut microbiota and the inflammatory response. Therefore, we consider it of great importance to include questions about probiotic and other supplement consumption in our FFQ in future studies to obtain more precise estimates and reduce the possibility of residual confounding.

## 5. Conclusions

Our findings suggest that a low RA of *A. muciniphila* increases the probability of presenting as high on the proinflammatory index when consuming a high intake of simple carbohydrates and saturated fats. This highlights the importance of developing prevention strategies based on modifiable factors in the pediatric population, such as dietary patterns, to avoid chronic low-grade inflammation and the associated comorbidities throughout one’s lifetime.

## Figures and Tables

**Table 1:** General characteristics of children according to the proinflammatory index.

Characteristics	Proinflammatory Index				*p*-Value
	Low *n* = 503 (50%)		High *n* = 503 (50%)		
	Median or Percentage ^1^	IQR ^2^	Median or Percentage ^1^	IQR ^2^	
Age (years)	9	2.5	9	3	**0.001**
**Sex**					
Girls (%)	51		49		0.626
Boys (%)	52		48		
Family history of obesity (%)	48		52		**0.003**
Family history of type 2 diabetes (%)	57		43		0.212
Total energy (Kcal)	2066.1	829.5	2065	1018.7	0.489
BMI (Kg/m^2^)	17.7	4.8	20.07	6.1	**<0.001**
Physical activity (METs)	2585.7	3122.1	2653.5	3318.7	0.733
*Akkermansia muciniphila* (RA)	0.0025	0.04	0.0033	0.07	0.084
**Cytokines**					
IL-6 (pg/mL)	20.5	63.0	32.2	169.8	**<0.001**
IL-10 (pg/mL)	9.4	49.6	7.8	34.6	**0.012**
TNF-α (pg/mL)	8.6	8.6	14.2	6.7	**<0.001**
Adiponectin (µg/mL)	5.7	1.5	4.6	0.9	**<0.001**

IQR, interquartile range; METs, metabolic equivalent of tasks; RA, relative abundance; IL-6, interleukin-6; IL-10, interleukin-10; TNF-α, tumoral necrosis factor alpha. ^1^ Values show medians or percentages and ^2^ interquartile range. Mann–Whitney U test for continuous variables or Chi-square for categorical variables. Statistically significant differences marked in bold *p* < 0.05.

**Table 2:** Association between *Akkermansia muciniphila* and proinflammatory index.

*A. muciniphila* ^1^	High Proinflammatory Index ^2^			
	*n*	OR	CI 95%	*p*-Value
High RA	335	1.00		
Medium RA	335	0.84	0.61–1.16	0.30
Low RA	336	0.86	0.62–1.18	0.36

RA, relative abundance. ^1^ Reference category: a high RA of *A. muciniphila*. ^2^ Multiple logistic regression adjusted for age, sex, physical activity, body mass index, family history of overweight–obesity, and dietary patterns.

**Table 3:** Association between intake of dietary patterns and being high on the proinflammatory index.

Tertiles of Intake ^1^	Dietary Patterns ^2^							
		Complex Carbohydrates and Protein ^1^				Simple Carbohydrates and Saturated Fats ^2^		
	*n*	OR	CI 95%	*p*-Value	*n*	OR	CI 95%	*p*-Value
Low	331	1.00			340	1.00		
Medium	346	0.92	0.66–1.27	0.62	329	0.85	0.61–1.18	0.34
High	329	1.03	0.75–1.43	0.81	337	0.83	0.58–1.15	0.27

^1^ Reference category: low intake. ^2^ Multiple logistic regression adjusted for age, sex, physical activity, body mass index, and family history of overweight–obesity.

**Table 4:** Joint effect of *Akkermansia muciniphila* and complex carbohydrates and protein intake on the odds of being high on the proinflammatory index.

*A. muciniphila* ^1^	Complex Carbohydrates and Protein ^2^											
	Low Intake				Medium Intake				High Intake			
	*n*	OR	CI 95%	*p*-Value	*n*	OR	CI 95%	*p*-Value	*n*	OR	CI 95%	*p*-Value
High RA	113	1.00			112	1.00			110	1.00		
Medium RA	115	0.75	0.43–1.32	0.32	119	0.92	0.53–1.62	0.79	101	0.93	0.52–1.68	0.83
Low RA	103	0.85	0.47–1.51	0.58	115	0.84	0.48–1.47	0.55	118	0.87	0.49–1.54	0.64

RA, relative abundance. ^1^ Reference category: a high RA of *A. muciniphila.* ^2^ Multiple logistic regression adjusted for age, sex, physical activity, body mass index, family history of overweight–obesity, and dietary pattern of simple carbohydrates and saturated fats.

**Table 5:** Joint effect of *Akkermansia muciniphila* and simple carbohydrates and saturated fats intake on the odds of being high on the proinflammatory index.

*A. muciniphila* ^1^	Simple Carbohydrates and Saturated Fats ^2^											
	Low Intake				Medium Intake				High Intake			
	*n*	OR	CI 95%	*p*-Value	*n*	OR	CI 95%	*p*-Value	*n*	OR	CI 95%	*p*-Value
High RA	114	1.00			122	1.00			99	1.00		
Medium RA	106	0.81	0.45–1.45	0.49	105	0.67	0.37–1.19	0.17	124	1.12	0.63–1.98	0.69
Low RA	120	0.51	0.29–0.89	**0.02**	102	0.79	0.45–1.41	0.44	114	1.63	0.90–2.94	**0.10**

RA, relative abundance. ^1^ Reference category: a high RA of *A. muciniphila.* ^2^ Multiple logistic regression adjusted for age, sex, physical activity, body mass index, family history of overweight–obesity, and dietary pattern of complex carbohydrates and protein. Statistically significant differences marked in bold *p* < 0.10.

## Data Availability

Data is contained within the article or Supplementary Material.

## Supplementary Materials

The following supporting information can be downloaded at: https://www.mdpi.com/article/10.3390/children10111799/s1, Table S1: Areas under the curve for each cytokine as predictors of the proinflammatory index; Table S2: Dietary patterns.

## List of Abbreviations

*A. muciniphila*	*Akkermansia muciniphila*
BMI	Body mass index
FFARs	Free fatty acids receptors
FFQ	Food frequency questionnaire
GalNAc	N-acetylgalactosamine
GlcNAc	N-acetylglucosamine
GLPs	Glucagon-like peptides
GPR	G-protein-coupled receptor
IL-10	Interleukin 10
IL-6	Interleukin 6
iNOS	Inducible nitric oxide synthase
LPSs	Lipopolysaccharides
METs	Metabolic equivalents
NO	Nitric oxide
PPARγ	Peroxisome proliferator-activated receptor gamma
q-PCR	Quantitative polymerase chain reaction
RA	Relative abundance
SCFAs	Short-chain fatty acids
T2D	Type 2 diabetes
TLR-4	Toll-like receptor 4
TNF-α	Tumor necrosis factor alpha
ZOs	Zonula occludens
