# Supplementary material for: Interaction between Akkermansia muciniphila and Diet Is Associated with Proinflammatory Index in School-Aged Children
Source: Children (Basel). 2023 Nov 10;10(11):1799. doi: 10.3390/children10111799 (PMC10670599; doi:10.3390/children10111799)
Supplement: Supplementary file 1 [file children-10-01799-s001.zip › children-2649763-supplementary.pdf]

Supplementary material

Table S1 Dietary patterns.

Food groups	Patterns [1]	
	Simple carbohydrates and saturated fats	Complex carbohydrates and protein
Fruits	-	0.4343
Vegetables	-	0.6473
Legumes	-	0.4837
Avocado	-	0.3506
Fish	-	0.4246
Chicken	-	0.3944
Red meat	0.3851	-
Sweet Breads	0.4465	-
Potato	-	0.4935
Flours	0.4611	-
Mexican dishes	0.4466	-
Fast food	0.362	-
Salty snack	0.6128	-
Candies	0.4791	-
Pork rind	0.4505	-
Sugary drinks	0.5433	-
Water	-0.4346	-
Contribution (%)	10.05	6.39

[1] Values show the loading factors that each food group contributed to the dietary patterns.

Table S2 Areas under the curve for each cytokine as predictors of the proinflammatory index.

Cytokines	Area under de curve [1]	CI 95%
IL-6 (pg/mL)	0.57	0.54-0.61
IL-10 (pg/mL)	0.80	0.77-0.82
TNF-α (pg/mL)	0.53	0.50-0.57
Adiponectin (μg/mL)	0.83	0.80-0.85

IL-6, interleukin-6; IL-10, interleukin-10; TNF-α, tumoral necrosis factor-alpha.
[1] Values show the discriminatory power of the proinflammatory index to classify children with inflammation correctly.
